# Therapist‐Guided Smartphone‐Based Aftercare for Inpatients With Severe Anorexia Nervosa (SMART‐AN): A Randomized Clinical Trial

**DOI:** 10.1002/eat.70142

**Published:** 2026-06-23

**Authors:** Sandra Schlegl, Andreas Gerich, Yannik Terhorst, Christina Fricke Neumayr, Ulrich Voderholzer

**Affiliations:** ^1^ Department of Psychiatry and Psychotherapy LMU University Hospital, LMU Munich Munich Germany; ^2^ Department of Clinical Psychology and Experimental Psychopathology Goethe‐University Frankfurt Germany; ^3^ Department of Psychological Methods and Assessment LMU Munich Munich Germany; ^4^ Schoen Clinic Roseneck Prien am Chiemsee Germany; ^5^ Department of Psychiatry and Psychotherapy University Hospital of Freiburg Freiburg Germany

**Keywords:** aftercare, anorexia nervosa, inpatient, RCT, smartphone, therapist‐guided

## Abstract

**Objective:**

To evaluate the efficacy of a 16‐week therapist‐guided, smartphone‐based aftercare intervention as an add‐on to treatment as usual (TAU) following inpatient treatment for anorexia nervosa (AN).

**Method:**

In this single‐center randomized clinical trial, 186 female inpatients with DSM‐5 AN (≥ 13 years; body mass index (BMI) ≥ 15.0 kg/m^2^ at discharge; smartphone access) were randomized to TAU plus smartphone‐based aftercare (intervention group, IG; *n* = 93) or TAU only (control group, CG; *n* = 93). The intervention used the *Recovery Record* app with individualized goals and therapist feedback of decreasing frequency over 16 weeks. Outcomes were assessed at discharge (T0), end of intervention (T1), and 6‐month follow‐up (T2) by blinded assessors, and were analyzed using linear mixed effect models (intention‐to‐treat). The primary outcome was the Eating Disorder Examination (EDE) global score; secondary outcomes included BMI, depressive symptoms, self‐reported eating disorder psychopathology, self‐efficacy, and stage of change.

**Results:**

At T1, there was no significant between‐group difference in EDE global score (mean difference, 0.12; 95% CI, −0.21 to 0.46), and results were similar at T2 (0.27; 95% CI, −0.13 to 0.67). Secondary outcomes did not differ clearly between groups. Intervention adherence was high (69.9% completed 16 weeks). Rehospitalizations occurred in 4.7% of participants by T1 and 26.4% by T2, without significant between‐group differences.

**Discussion:**

In a context of intensive TAU with rapid access to outpatient psychotherapy, adding therapist‐guided smartphone‐based aftercare did not yield additional short‐term symptom benefit after inpatient treatment for AN. Future work should refine digital aftercare and embed it more closely into blended treatment models.

**Trial Registration:** ClinicalTrials.gov identifier: NCT04228939

## Summary

- In a randomized clinical trial of 186 females with anorexia nervosa, a 16‐week therapist‐guided smartphone‐based aftercare program did not reduce eating disorder psychopathology beyond intensive treatment as usual.
- Uptake of outpatient psychotherapy and medical follow‐up after discharge was high in both groups, yet rehospitalization remained clinically relevant during follow‐up, with more than one quarter of participants rehospitalized by the 6‐month follow‐up after the 4‐month aftercare period.
- Smartphone‐based aftercare appears feasible and safe, but future interventions may need greater intensity and closer integration with outpatient psychotherapy to achieve clinically meaningful benefits.

## 1 Introduction

Anorexia nervosa (AN) is a serious mental disorder characterized by a restriction of energy intake leading to significantly low body weight, an intense fear of gaining weight or becoming fat despite being underweight, and a disturbance in the way one's own body weight or shape is experienced (American Psychiatric Association 2013). Lifetime prevalence in women ranges from 0.6% to 6.1% worldwide (Silén and Keski‐Rahkonen 2022). Despite evidence‐based psychotherapies, treatment efficacy remains modest, and AN is associated with high premature mortality and low long‐term recovery rates (Phillipou et al. 2025).

Inpatient treatment demonstrates medium‐to‐large effects on weight and symptom improvement, but outcomes are often insufficient: about half of patients do not achieve a body mass index (BMI) ≥ 17.5 kg/m^2^ at discharge, and relapse rates of up to 41% have been reported (Carter et al. 2012; Schlegl et al. 2016, 2014). The period following discharge from inpatient treatment is a particularly vulnerable phase for patients with AN. Although inpatient care can produce meaningful short‐term improvements in weight and symptoms, many patients struggle to maintain these gains once they return to everyday life, where meal support, structure, and clinical monitoring are markedly reduced. This transition may increase the risk of symptom deterioration, relapse, and readmission, highlighting the need for accessible aftercare interventions that can help bridge the gap between inpatient and community‐based treatment.

As Zipfel et al. (2022) emphasized, optimal care pathways, especially for adults with AN, should include interventions that bridge the transition from inpatient services to home‐based care and strengthen community support. Digital mental health interventions (DMHI) may help close this gap by providing structured, accessible, and scalable aftercare.

Early work summarized by Schlegl et al. (2015) identified 40 technology‐based interventions for eating disorders (EDs). Only three specifically addressed relapse prevention—an internet‐based program for AN and two SMS‐based interventions for bulimia nervosa. While relying on earlier technologies, these studies nonetheless showed promising results. More recently, Giel et al. (2021) reviewed post‐inpatient interventions and highlighted the potential of digital formats for relapse prevention, including the ongoing SUSTAIN trial, a videoconference‐based psychotherapy protocol for AN, and the multicenter TRIANGLE trial. The latter has since been completed and tested ECHOMANTRA, a digital guided self‐management program for patients and carers during the transition from intensive to community care, but did not demonstrate superiority over treatment as usual (TAU) (Cardi et al. 2024).

Smartphone‐based applications have since become a central focus within DMHI research and are increasingly investigated in EDs (Cruz et al. 2025; Fairburn and Rothwell 2015; Juarascio et al. 2015; O'Leary and Torous 2022; Wasil et al. 2021). Within this line of work, Fitzsimmons‐Craft et al. (2023) are piloting a cognitive‐behavioral therapy (CBT)‐based self‐help mobile app for post‐acute treatment of AN.

*Recovery Record* is the most widely used smartphone application specifically developed for EDs. It integrates principles from evidence‐based psychotherapies (Juarascio et al. 2015) and has demonstrated feasibility, acceptability, and meaningful use across diagnoses and treatment settings (Keshen et al. 2020; Lindgreen et al. 2021; Reilly et al. 2024; Tregarthen et al. 2019).

Meta‐analyses provide further support for digital interventions. Smartphone‐based interventions in other psychiatric disorders show small‐to‐moderate benefits, particularly when CBT‐based and therapist‐guided (Linardon et al. 2019), supporting their potential relevance for ED aftercare. In addition, a recent meta‐analysis demonstrated small but significant effects of digital interventions in inpatient settings, especially for blended treatments and post‐treatment aftercare (Diel et al. 2024).

Building on this evidence, *Recovery Record* was first translated and implemented in Germany in collaboration with Recovery Record Inc. For our pilot randomized clinical trial (RCT) (*N* = 40), the company programmed additional features accessible only via a study code such as weekly coping strategies and goals tailored to the aftercare context. The intervention showed high adherence and acceptability, along with preliminary evidence of benefit, although effects diminished at follow‐up (FU) (Neumayr et al. 2019).

The present study builds directly on this pilot and responds to the call by O'Leary and Torous (2022) for translational research on digital tools for EDs.

### 1.1 Objectives

The aim of this RCT was to evaluate the efficacy of a therapist‐guided, smartphone‐based aftercare intervention as an add‐on to TAU following inpatient treatment for AN. The primary hypothesis was that, at the end of the intervention (T1), participants in the intervention group (IG) would show lower overall ED psychopathology compared with TAU. Secondary hypotheses included lower ED psychopathology at 6‐month FU (T2), higher BMI, greater reductions in depressive symptoms, higher stage of change, and lower rehospitalization rates at T1 and T2.

## 2 Method

Reporting follows current CONSORT recommendations for RCTs (Hopewell et al. 2025).

### 2.1 Patient and Public Involvement

At the time of trial design and conduct, patient and public involvement was not yet routinely integrated into clinical trial protocols. Accordingly, there was no formal patient or public involvement in the design, conduct, or reporting of this study.

### 2.2 Trial Design

This single‐center, prospective RCT used a parallel‐group design with 1:1 allocation and was planned as a superiority design. Ethical approval was obtained from the ethics committee of the Medical Faculty of Ludwig Maximilian University (LMU) Munich. The trial was pre‐registered at ClinicalTrials.gov (NCT04228939), and the study protocol was published (Schlegl et al. 2020). As described in the study protocol, the a priori sample size was based on the effect size observed in our pilot RCT (Neumayr et al. 2019), which indicated a medium between‐group effect (*d* = 0.50) on the primary outcome. Assuming *α* = 0.05, power = 0.80, an AR(1) correlation of 0.30, and 25% attrition between timepoints, the required sample size was *N* = 186. Randomization was conducted using randomizer.org by an independent researcher not involved in assessments. Outcome assessors were blinded to group allocation (single‐blind design).

### 2.3 Trial Setting

Patients were recruited at Schoen Clinic Roseneck, a specialized inpatient ED unit in Germany. After discharge, all participants received TAU, defined as naturalistic post‐discharge care arranged with routine community providers. In the German routine care context, this typically includes face‐to‐face outpatient psychotherapy, with medical and/or psychiatric follow‐up appointments provided where indicated. Outpatient psychotherapy is commonly delivered in 50‐min sessions, often on a weekly basis. Within the present trial, the specific intensity and therapeutic orientation of TAU were not standardized and reflected routine care, most commonly cognitive‐behavioral or psychodynamic approaches. Depending on clinical need, TAU could also include weight monitoring, nutritional counseling, day treatment, or rehospitalization. TAU utilization was documented at T1 and T2.

Participants randomized to the IG received, in addition to TAU, the therapist‐guided aftercare delivered digitally via the *Recovery Record* app.

### 2.4 Eligibility Criteria

Eligibility criteria included a primary diagnosis of AN (DSM‐5: 307.1) at admission, as assessed by diagnostically relevant items of the Eating Disorder Examination Interview (EDE) (Hilbert and Tuschen‐Caffier 2016a), female sex (to ensure a more homogeneous sample and because the eligible inpatient population with AN at the recruitment site during the study period was overwhelmingly female), and age ≥ 13 years. Additional requirements were regular completion of inpatient treatment (defined as a planned discharge after completion of the intended inpatient treatment program rather than premature termination of treatment), a minimum weight gain during admission (≥ 2 BMI points if BMI at admission < 14 or ≥ 1 BMI point if BMI at admission ≥ 14) (Fichter et al. 2012), ownership of a smartphone, and provision of informed consent (with parental consent required for minors). Patients were excluded if they had a Beck Depression Inventory‐II (BDI‐II) (Hautzinger et al. 2009) score > 29 at discharge or suicidal tendency (Item 9 of the BDI‐II > 1 at discharge) because the intervention was designed as a low‐intensity digital aftercare adjunct for clinically stabilized patients. Severe depressive symptoms, particularly when accompanied by suicidal ideation, were considered to indicate a need for more intensive psychiatric monitoring and treatment than could be provided within the study intervention. Furthermore, patients who required a very high level of continuing care after inpatient treatment (e.g., therapeutic living community or day clinic) were excluded.

### 2.5 Intervention and Comparator

Patients randomized to the IG received a therapist‐guided smartphone‐based aftercare intervention for 16 weeks. They were invited to download the German version of the *Recovery Record* app and to link with an aftercare therapist. After discharge, they were instructed to monitor meals, emotions, cognitions, and symptoms for 16 weeks. The aftercare therapist set individualized post‐discharge goals and suggested coping strategies. Individualized therapist feedback was provided in‐app twice weekly during weeks 1–4, once weekly during weeks 5–8, every other week during weeks 9–12, and once in week 16. This approach was consistent with the principle of minimum intervention (Kanfer et al. 2011). *Recovery Record* is a CBT‐based smartphone application that also integrates elements of dialectical behavior therapy, acceptance and commitment therapy, and motivational enhancement (Tregarthen et al. 2015). The German version used in this trial included additional features specifically tailored for post‐discharge aftercare, which were developed and tested in our pilot RCT (Neumayr et al. 2019).

Patients randomized to the control group (CG) received TAU, defined as naturalistic post‐discharge care arranged with routine providers (e.g., outpatient psychotherapy, medical and psychiatric follow‐up, weight monitoring, nutritional and counseling services, and, if needed, higher‐intensity care such as day treatment or rehospitalization). TAU was documented at T1 and T2.

### 2.6 Privacy and Data Security

Several steps were taken to ensure that patient data were handled securely and in accordance with ethical and legal standards. All data transmitted through the app were encrypted in transit (Transport Layer Security) and at rest (Advanced Encryption Standard). A password‐protected database was regularly backed up and maintained on a secure server located in Frankfurt, Germany. *Recovery Record* operates a European compliance region with infrastructure located in Germany and processes European user data in accordance with applicable European data protection requirements, including the EU General Data Protection Regulation (GDPR).

### 2.7 Outcomes

The primary outcome was ED psychopathology, assessed with the EDE global score at T1. Secondary outcomes included EDE subscales, BMI (measured by nurses at T0 and by general practitioners at T1 and T2), the Eating Disorder Examination‐Questionnaire (EDE‐Q) (Hilbert and Tuschen‐Caffier 2016b), the BDI‐II, the Stages of Change Questionnaire for Eating Disorders (SOCQ‐ED) (von Brachel et al. 2012), and the General Self‐Efficacy Scale (GSE) (Schwarzer and Jerusalem 1999). Assessments were conducted at discharge (baseline, T0), after 16 weeks (end of intervention, T1), and at 6‐month FU (T2). At each timepoint, blinded assessors conducted structured clinical interviews, and patients completed online questionnaires (via the Unipark portal). A self‐developed questionnaire assessed qualitative evaluation of the app at T1, and health care utilization was recorded at T1 and T2. Patients received €60 for completing all three assessments. Sociodemographic and clinical characteristics, for example, illness duration, and previous treatments, were obtained from medical records.

Internal consistency in the present sample was assessed via Cronbach's alpha and was excellent (> 0.90) for the EDE, EDE‐Q, BDI‐II, and GSE, whereas it was good for the SOCQ‐ED (0.82).

### 2.8 Changes to Trial Protocol

For our analyses, we used R rather than SPSS to improve transparency and reproducibility, as it is open‐source and free. This software change was not expected to materially affect the results (Baker et al. 2024). We retained the standard lme4 specification with random intercepts and independent residual errors for reasons of parsimony and generalizability, and because lme4 does not support user‐specified residual covariance structures. This modeling choice was not expected to meaningfully affect the model outcomes (Charles and John 2011; Kincaid 2005).

Between‐group effect sizes were derived from differences in marginal means divided by the model residual standard deviation (SD) rather than the pooled baseline SD. Finally, for exploratory purposes, we reported timepoint‐specific contrasts to aid interpretation, irrespective of omnibus test significance.

### 2.9 Harms

Adverse events (AEs) and serious adverse events (SAEs) were systematically monitored throughout the trial, independent of their presumed relation to the intervention. SAEs were defined as death, life‐threatening events, or hospitalization; rehospitalizations were therefore recorded as SAEs.

### 2.10 Statistical Methods

Analyses were conducted in R version 4.4.0 (R Core Team 2025). Internal consistency of the outcome measures was assessed via Cronbach's alpha at baseline.

The primary outcome (EDE global score) was analyzed with linear mixed effect models (LMMs) (lme4, lmerTest) including fixed effects for time (T0, T1, T2), group (IG vs. CG), and their interaction, and a random intercept for participants. This approach allowed estimation of adjusted group differences at the prespecified primary endpoint (T1) as well as at follow‐up (T2). A quadratic time term was explored but not retained because it did not improve model fit based on the Akaike information criterion. Normality of residuals was assessed using Q–Q plots. Estimated marginal means (EMMs) and pairwise group contrasts were obtained for each timepoint with the emmeans package, and 2‐sided Wald tests with *α* = 0.05 and 95% confidence intervals (CIs) were reported. Within‐group changes from T0 to T1 and T0 to T2 were examined exploratorily with one‐sample *t*‐tests (*α* = 0.05) and corresponding effect sizes.

Primary analyses followed the intention‐to‐treat principle and were based on observed data.

Sensitivity analyses additionally included multiple imputation, a per‐protocol analysis restricted to participants who completed all 16 intervention weeks, and an adherence‐based analysis using the ≥ 12‐week adherence sample, by which point 14 of 15 scheduled therapist feedback contacts had been delivered. Missingness at random was assumed for missing data for all analyses.

Secondary outcomes were analyzed with the same LMM structure.

## 3 Results

### 3.1 Participant Flow

Of 519 patients admitted during the study period, 428 were screened and 186 provided written informed consent and were randomized to the IG (*n* = 93) or CG (*n* = 93) (see Figure 1).

**FIGURE 1:**
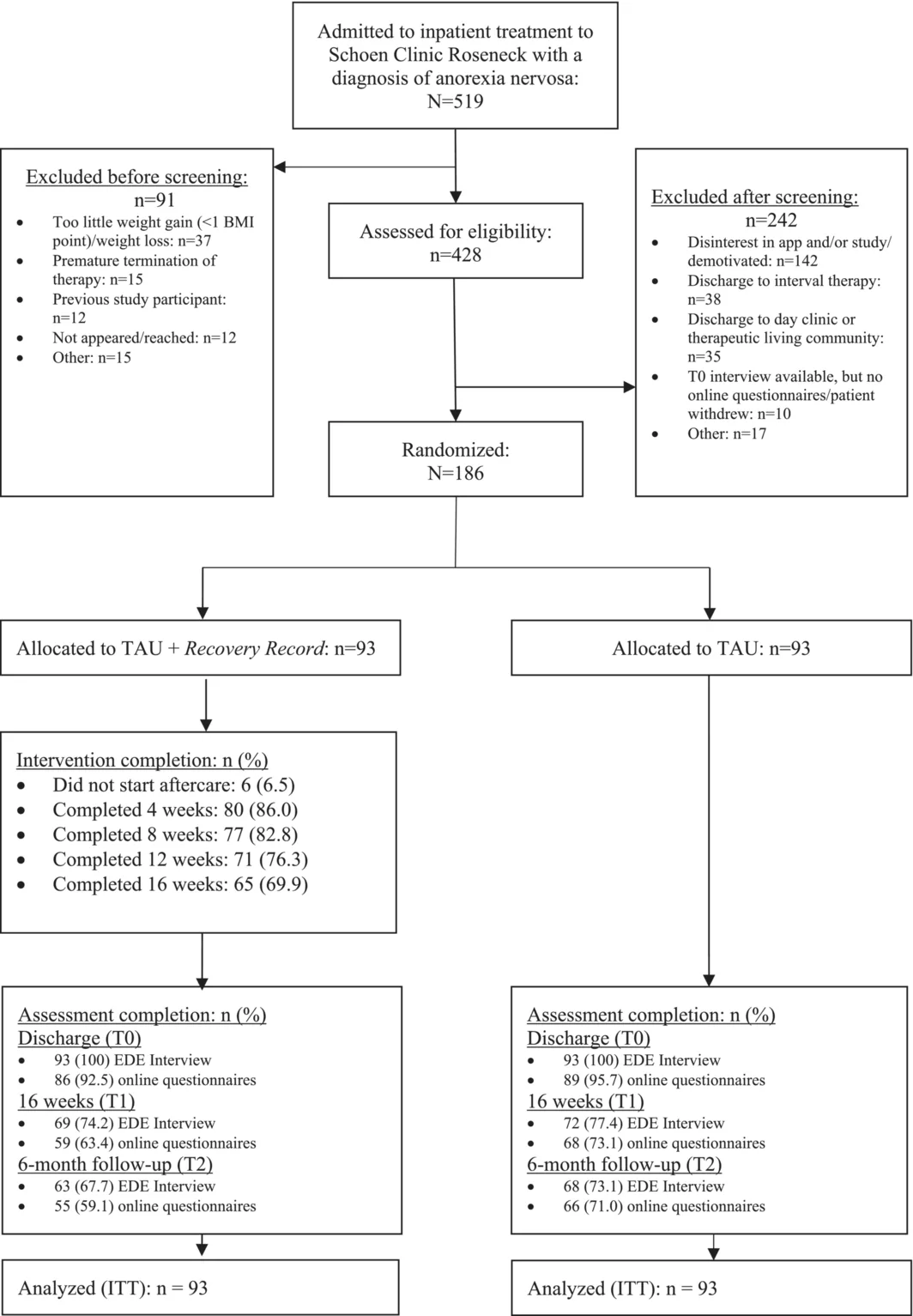
Flow chart of recruitment of the randomized clinical trial. BMI, body mass index; EDE, Eating Disorder Examination; ITT, intention‐to‐treat; TAU, treatment as usual.

### 3.2 Recruitment

The SMART‐AN single‐center RCT was conducted from January 2019 to December 2021.

### 3.3 Intervention and Comparator Delivery

The intervention was delivered as planned in the IG as an adjunct to TAU. TAU itself was provided in both groups by community providers. In the IG, adherence was high, with 86% completing 4 weeks, 82.8% completing 8 weeks, 76.3% completing 12 weeks, and 69.9% completing the full 16 weeks of aftercare. Fidelity was supported through therapist training prior to study start and monthly supervision. Post‐discharge health care utilization was high and largely comparable between groups. At T1, 94.1% of patients in the CG and 89.8% in the IG received outpatient psychotherapy; at T2, rates were 86.4% in the CG and 92.7% in the IG, respectively. Inpatient treatment after discharge was less common: at T1, 1.4% of CG patients and 8.5% of IG patients reported further inpatient care (overall 4.7%); by T2, this had increased to 25.8% in the CG and 27.3% in the IG (overall 26.4%) (see Table S1). These findings indicate that both groups had substantial access to routine post‐discharge care in addition to the study intervention in the IG.

### 3.4 Baseline Data

Table 1 presents the sociodemographic and clinical variables of the IG and CG.

**TABLE 1:** Participant characteristics (*N* = 186).

Baseline variable	Intervention group (*n* = 93)	Control group (*n* = 93)
Age in years *M* (SD), range	19.69 (6.56), 13–49	19.49 (6.52), 13–51
Adolescents *n* (%)	48 (51.6)	47 (50.5)
Weight‐related characteristics *M* (SD)		
BMI in kg/m^2^ (at admission)	15.48 (1.60)	15.26 (1.68)
Percentile (at admission)	1.76 (3.38)	1.82 (5.44)
SDS (at admission)	−3.12 (1.40)	−3.05 (3.07)
BMI in kg/m^2^ (at randomization/baseline)	18.84 (1.40)	18.87 (1.24)
Percentile (at randomization/baseline)	19.89 (14.28)	20.06 (15.41)
SDS (at randomization/baseline)	−1.03 (0.70)	−1.00 (0.66)
Minimal weight in kg	40.47 (5.89)	39.75 (5.56)
Minimal BMI in kg/m^2^	14.18 (2.22)	14.26 (1.63)
Maximal weight in kg	60.51 (10.50)	57.59 (7.27)
Maximal BMI in kg/m^2^	21.89 (6.08)	20.64 (2.35)
Weight suppression in kg	20.11 (9.50)	17.79 (6.57)
BMI severity (at admission) *n* (%)		
Mild	19 (20.4)	12 (12.9)
Moderate	18 (19.4)	18 (19.4)
Severe	20 (21.5)	25 (26.9)
Extreme	36 (38.7)	38 (40.9)
BMI severity (at discharge) *n* (%)		
Mild	83 (89.3)	89 (95.7)
Moderate	8 (8.6)	3 (3.2)
Severe	3 (3.2)	2 (2.2)
Extreme	0 (0.0)	0 (0.0)
Duration of ED in years *M* (SD)	4.17 (5.08)	3.62 (3.53)
Inpatient treatment duration in days *M* (SD)	122.30 (51.78)	120.05 (52.13)
Previous inpatient treatment *M* (SD)	1.32 (1.92)	1.15 (1.72)
Previous outpatient treatment *M* (SD)	1.21 (1.17)	1.15 (1.59)
Subtype of AN *n* (%)		
Restrictive type (F50.00)	54 (58.1)	74 (79.6)
Binge‐eating/purging type (F50.01)	35 (37.6)	13 (14.0)
Atypical AN (F50.1)	4 (4.3)	5 (5.4)
Any comorbid disorder *n* (%)	73 (78.5)	66 (71.0)
Comorbidity^a^ *n* (%)		
Mild depressive episode (F32.0)	6 (6.5)	2 (2.2)
Moderate depressive episode (F32.1)	33 (35.5)	33 (35.5)
Severe depressive episode (F32.2)	9 (9.7)	13 (14.0)
Recurrent moderate depressive disorder (F33.1)	11 (11.8)	8 (8.6)
Recurrent severe depressive disorder (F33.2)	7 (7.5)	5 (5.4)
Mixed obsessional thoughts and acts (F42.2)	7 (7.5)	1 (1.1)
Posttraumatic stress disorder (F43.1)	8 (8.6)	1 (1.1)
EDE *M* (SD)		
Global Score	2.04 (1.20)	1.99 (1.14)
Restraint	1.23 (1.16)	1.14 (1.05)
Eating concern	1.14 (0.97)	1.17 (1.09)
Weight concern	2.13 (1.48)	2.05 (1.38)
Shape concern	2.98 (1.61)	2.87 (1.53)
EDE‐Q *M* (SD)		
Global Score	2.27 (1.31)	2.02 (1.25)
Restraint	1.41 (1.15)	1.25 (1.17)
Eating concern	1.48 (1.21)	1.29 (1.10)
Weight concern	2.33 (1.57)	2.00 (1.50)
Shape concern	3.27 (1.74)	2.98 (1.65)
BDI‐II Global Score *M* (SD)	19.50 (13.40)	15.42 (12.07)
GSE Global Score *M* (SD)	24.33 (5.92)	26.27 (5.40)
SOCQ‐ED Global Score *M* (SD)	61.77 (8.78)	63.09 (9.49)

Abbreviations: AN, anorexia nervosa; BDI‐II, Beck Depression Inventory‐II; BMI, body mass index; ED, eating disorder; EDE, Eating Disorder Examination; EDE‐Q, Eating Disorder Examination‐Questionnaire; GSE, General Self‐Efficacy Scale; SDS, Standard Deviation Score; SOCQ‐ED, Stages of Change Questionnaire for Eating Disorders.

^a^ Only diagnoses occurring in more than 5% of participants in either group are shown.

Of the 186 randomized participants, 141 (75.8%) provided primary outcome data at T1 and 131 (70.4%) at T2. Baseline characteristics by assessment completion status at 16 weeks and 6‐month FU are shown in Tables S2 and S3. Questionnaire data (self‐report measures) were available for 127 (68.3%) participants at T1 and 121 (65.1%) at T2.

### 3.5 Efficacy Analyses

#### 3.5.1 Primary Outcome

The LMM revealed no statistically significant group × time interaction on the EDE global score (estimate = 0.02, SE = 0.02, *t*(286.05) = 1.40, *p* = 0.163, Table 2). Consistent with this, there was no statistically significant adjusted between‐group difference at the prespecified primary endpoint (T1) (mean difference, 0.12; 95% CI, −0.21 to 0.46; *p* = 0.47, Table 3). Additional descriptive timepoint‐specific group comparisons are provided in Table 3. Exploratory within‐group tests showed no meaningful change in EDE global scores from T0 to T1 or from T0 to T2 in either group (see Table S6). The descriptive trajectories of EMMs are illustrated in Figure 2.

**TABLE 2:** Fixed effects estimates from linear mixed effect models.

Outcome	Fixed effects	Estimate	SE	df	*t* value	*p* value
*Primary outcome*						
EDE Global Score	Intercept	2.02	0.13	243.41	15.83	< 0.001***
	Timepoint	−0.01	0.01	285.73	−1.13	0.258
	Group	0.02	0.18	241.84	0.13	0.895
	Timepoint:Group	0.02	0.02	286.05	1.40	0.163
*Secondary outcomes*						
EDE‐Q Global Score	Intercept	2.10	0.14	223.99	14.61	< 0.001***
	Timepoint	0.01	0.01	261.91	0.76	0.446
	Group	0.15	0.21	225.49	0.75	0.452
	Timepoint:Group	0.03	0.02	264.29	1.46	0.145
BMI	Intercept	18.78	0.17	267.89	112.02	< 0.001***
	Timepoint	−0.05	0.02	255.98	−2.43	0.016*
	Group	0.01	0.24	268.42	0.02	0.981
	Timepoint:Group	−0.00	0.03	256.31	−0.08	0.938
BDI‐II Global Score	Intercept	16.35	1.40	212.97	11.75	< 0.001***
	Timepoint	0.47	0.12	258.00	4.05	< 0.001***
	Group	3.49	1.99	214.20	1.76	0.081
	Timepoint:Group	0.25	0.17	259.93	1.43	0.155
GSE Global Score	Intercept	25.94	0.61	227.99	42.63	< 0.001***
	Timepoint	−0.10	0.06	262.72	−1.74	0.082
	Group	−1.58	0.87	229.60	−1.81	0.071
	Timepoint:Group	−0.04	0.09	265.30	−0.41	0.685
SOCQ‐ED Global Score	Intercept	62.15	1.12	242.13	55.60	< 0.001***
	Timepoint	−0.19	0.12	268.60	−1.66	0.097
	Group	−0.66	1.60	243.98	−0.41	0.678
	Timepoint:Group	−0.19	0.17	271.63	−1.13	0.258

Abbreviations: BDI‐II, Beck Depression Inventory‐II; BMI, body mass index; df, degrees of freedom; EDE, Eating Disorder Examination; EDE‐Q, Eating Disorder Examination‐Questionnaire; GSE, General Self‐Efficacy Scale; SE, standard error; SOCQ‐ED, Stages of Change Questionnaire for Eating Disorders.

* *p* < 0.05.

** *p* < 0.01.

*** *p* < 0.001.

**TABLE 3:** Estimated marginal means and group comparisons across timepoints.

Outcome	Timepoint	Intervention group EMM (95% CI) [*n*]	Control group EMM (95% CI) [*n*]	Difference in LS means (95% CI)	*p*	Effect size (95% CI)
Primary outcome						
EDE Global Score	Baseline	2.05 (1.80–2.30) [93]	2.02 (1.77–2.28) [90]	0.02 (−0.33–0.38)	0.895	0.03 (−0.45–0.52)
	16 weeks	2.09 (1.86–2.33) [69]	1.97 (1.73–2.20) [71]	0.12 (−0.21–0.46)	0.467	0.17 (−0.29–0.62)
	6‐month FU	2.16 (1.87–2.44) [62]	1.88 (1.60–2.16) [67]	0.27 (−0.13–0.67)	0.181	0.37 (−0.17–0.92)
Secondary outcomes						
EDE‐Q Global Score	Baseline	2.25 (1.96–2.54) [86]	2.10 (1.82–2.38) [89]	0.15 (−0.25–0.56)	0.452	0.20 (−0.32–0.71)
	16 weeks	2.41 (2.14–2.68) [59]	2.14 (1.87–2.41) [68]	0.27 (−0.11–0.65)	0.164	0.34 (−0.14–0.83)
	6‐month FU	2.64 (2.31–2.97) [55]	2.20 (1.89–2.51) [66]	0.44 (−0.01–0.90)	0.056	0.56 (−0.02–1.14)
BMI	Baseline	18.79 (18.46–19.12) [93]	18.78 (18.45–19.11) [93]	0.01 (−0.46–0.47)	0.981	0.00 (−0.39–0.40)
	16 weeks	18.57 (18.27–18.88) [54]	18.58 (18.28–18.88) [56]	0.00 (−0.43–0.42)	0.986	0.00 (−0.36–0.36)
	6‐month FU	18.25 (17.84–18.66) [54]	18.27 (17.86–18.68) [55]	−0.02 (−0.59–0.56)	0.952	−0.01 (−0.50–0.47)
BDI‐II Global Score	Baseline	19.84 (17.05–22.64) [86]	16.35 (13.61–19.10) [89]	3.49 (−0.43–7.41)	0.081	0.51 (−0.06–1.07)
	16 weeks	22.74 (20.06–25.41) [59]	18.25 (15.64–20.87) [68]	4.48 (0.75–8.22)	0.019	0.65 (0.11–1.19)
	6‐month FU	27.07 (23.94–30.20) [55]	21.11 (18.12–24.09) [66]	5.96 (1.64–10.28)	0.007	0.86 (0.24–1.49)
GSE Global Score	Baseline	24.36 (23.14–25.59) [86]	25.94 (24.74–27.14) [89]	−1.58 (−3.29–0.14)	0.071	−0.46 (−0.95–0.04)
	16 weeks	23.81 (22.67–24.96) [59]	25.53 (24.41–26.65) [68]	−1.72 (−3.32 to −0.11)	0.036	−0.50 (−0.96 to −0.03)
	6‐month FU	22.99 (21.59–24.39) [55]	24.92 (23.59–26.25) [66]	−1.93 (−3.86–0.01)	0.051	−0.56 (−1.12–0.00)
SOCQ‐ED Global Score	Baseline	61.48 (59.23–63.73) [86]	62.15 (59.95–64.35) [89]	−0.66 (−3.81–2.48)	0.678	−0.10 (−0.55–0.36)
	16 weeks	59.94 (57.86–62.02) [59]	61.38 (59.35–63.41) [68]	−1.44 (−4.35–1.47)	0.331	−0.21 (−0.63–0.21)
	6‐month FU	57.62 (55.00–60.24) [55]	60.22 (57.75–62.69) [66]	−2.60 (−6.20–1.00)	0.157	−0.38 (−0.90–0.15)

Abbreviations: BDI‐II, Beck Depression Inventory‐II; BMI, body mass index; CI, confidence interval; EDE, Eating Disorder Examination; EDE‐Q, Eating Disorder Examination‐Questionnaire; EMM, estimated marginal means; FU, Follow‐Up; GSE, General Self‐Efficacy Scale; LS, Least Squares; SOCQ‐ED, Stages of Change Questionnaire for Eating Disorders.

**FIGURE 2:**
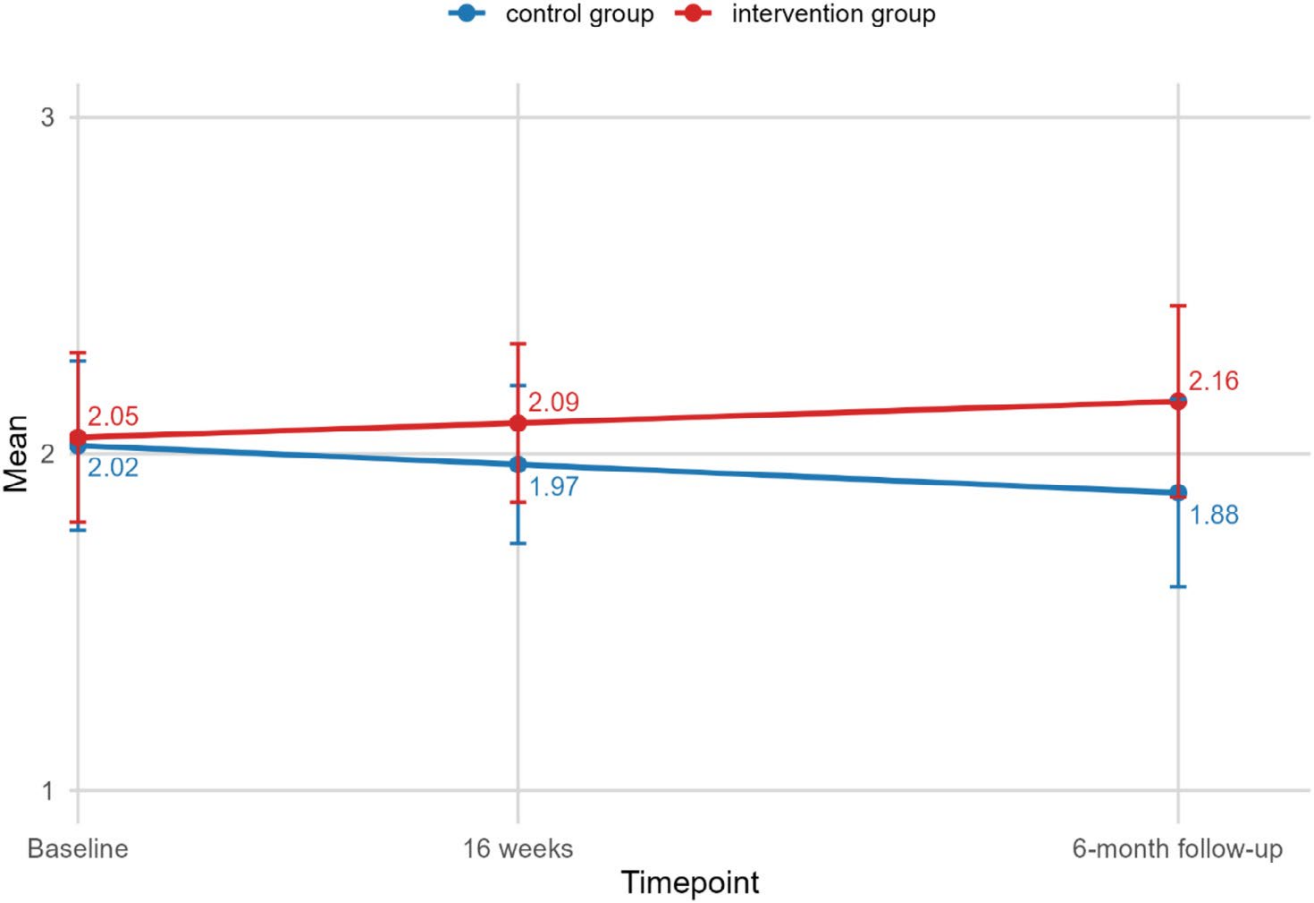
Severity of eating disorder psychopathology assessed by the Eating Disorder Examination (global score) across timepoints (baseline = discharge, 16 weeks, 6‐month follow‐up) and study groups (intervention group, control group). Values represent estimated marginal means (±95% CIs) from intention‐to‐treat analyses using linear mixed effect models.

#### 3.5.2 Secondary Outcomes

For the secondary outcomes, the LMMs showed no statistically significant group × time interactions (Table 2). Timepoint‐specific between‐group differences were observed for depressive symptoms (BDI‐II) and general self‐efficacy (GSE), but these findings should be interpreted cautiously because the corresponding interaction effects were not significant and the IG had already shown less favorable baseline values on these measures. Additional descriptive timepoint‐specific group comparisons for the secondary outcomes are presented in Table 3. Results for secondary outcome subscales are reported in Table S4 (fixed effects estimates from the linear mixed effect models) and Table S5 (estimated marginal means and timepoint‐specific group comparisons), and within‐group mean changes with corresponding effect sizes are provided in Table S6.

### 3.6 Sensitivity Analyses

Sensitivity analyses using multiple imputation, the per‐protocol population, and the ≥ 12‐week adherence sample yielded results consistent with the primary analyses, suggesting that missing data and deviations from the intended intervention had minimal impact on the overall pattern of findings (see Tables S7–S8).

### 3.7 Harms

At T1, 6 of 127 participants with available data (4.7%) reported further inpatient treatment (IG 5/59 [8.5%], CG 1/68 [1.4%]). By T2, this had increased to 32 of 121 participants (26.4%) (IG 15/55 [27.3%], CG 17/66 [25.8%]). No statistically significant between‐group differences were observed at either timepoint. There was no indication that these inpatient readmissions were related to the study intervention. Readmissions were not based on study‐specific criteria but reflected routine clinical care decisions made independently by the treating services. In general, rehospitalization indicated clinically significant deterioration requiring a higher level of care, such as renewed weight loss/medical instability, worsening ED psychopathology, or other psychiatric indications requiring inpatient treatment. No other AEs or SAEs occurred.

## 4 Discussion

This RCT tested whether adding a therapist‐guided, smartphone‐based aftercare intervention to TAU after inpatient treatment for AN would improve outcomes. The findings indicate that the intervention did not provide measurable additional benefit on ED psychopathology or secondary outcomes at 16 weeks or 6 months.

One possible explanation is that participants in both groups received intensive follow‐up care after discharge, including rapid initiation of outpatient psychotherapy and medical/psychiatric monitoring. Importantly, TAU uptake was high in both groups, with most participants receiving outpatient psychotherapy after discharge. This is relevant for interpreting the present findings, because the intervention was tested not against absent or minimal follow‐up care, but against a context of substantial routine post‐discharge treatment. Although TAU was intensive, we had expected that the smartphone‐based intervention might still provide incremental benefit because the post‐discharge period in AN is characterized by substantial relapse risk and challenges in maintaining treatment gains in everyday life. The intervention was designed to bridge this transition by extending support beyond inpatient care through structured self‐monitoring, individualized goals, and therapist guidance between routine outpatient contacts. At the same time, the post‐discharge phase remained clinically vulnerable, as reflected in worsening depressive symptoms and substantial rehospitalization rates across both groups. In this context, the therapist‐guided smartphone intervention may not have provided sufficient additional benefit beyond TAU to influence outcomes measurably. Moreover, because relapse risk in AN extends well beyond the immediate post‐discharge period, the 16‐week intervention and 6‐month follow‐up may have been too short to capture longer‐term effects. The present findings should also be interpreted in the context of prior research on digital interventions in EDs and related mental health conditions. In AN specifically, our results are in line with the TRIANGLE trial, which likewise did not find clear superiority of a guided digital transition intervention over TAU during the step‐down phase from intensive to community care (Cardi et al. 2024). More broadly, digital interventions in EDs appear feasible and acceptable, but evidence for clinical benefit remains mixed and may depend on factors such as treatment intensity, degree of guidance, and integration into ongoing care (Cruz et al. 2025; Giel et al. 2021; O'Leary and Torous 2022). A similar pattern has been described in other psychiatric conditions, where stronger effects are more likely when digital interventions are therapist‐guided, CBT‐based, and, in some cases, for interventions that are more closely integrated into ongoing care (Linardon et al. 2019; Diel et al. 2024).

The intervention may also have been too weakly integrated. It was delivered as an adjunct alongside outpatient psychotherapy and medical/psychiatric follow‐up, but it was not formally embedded in ongoing outpatient treatment. Outpatient therapists were not systematically involved, and the smartphone‐based feedback was not coordinated with the patient's evolving face‐to‐face treatment plan. In contrast, more strongly blended care models may yield greater benefit. Furthermore, engagement declined over time, reducing exposure to self‐monitoring and feedback and thereby likely attenuating the intervention's effectiveness, a pattern consistent with the well‐documented problem of declining engagement and early attrition in digital interventions (Boucher and Raiker 2024). Finally, although therapist feedback in the app was individualized, it followed a structured framework and was not necessarily aligned with each patient's evolving outpatient treatment plan.

Strengths include the randomized, assessor‐blinded design, adequate power, inclusion of adolescents and adults, and systematic monitoring of app use (detailed engagement analyses will be reported elsewhere). Rehospitalizations were observed but not considered related to the intervention.

Several limitations should be considered. The sample included only female patients, limiting generalizability to male patients with AN. Despite randomization, the IG showed a somewhat less favorable baseline profile, including higher depressive symptoms, lower self‐efficacy, and a higher proportion of patients with the binge‐purge subtype; although these imbalances likely occurred by chance, they should be considered when interpreting secondary outcomes, particularly BDI‐II and GSE. Engagement with the app declined over time, which may have reduced exposure to key intervention components. In addition, the 16‐week intervention and 6‐month follow‐up may have been too short to capture longer‐term effects, given the enduring relapse risk in AN. Although the trial was powered to detect a medium‐sized incremental benefit above TAU, small additional effects cannot be ruled out. Interrater reliability for the EDE interviews was not formally assessed. Finally, the single‐center setting and the unusually rapid access to outpatient psychotherapy and medical follow‐up may limit generalizability to other care contexts.

In summary, in a context with rapid access to outpatient psychotherapy and medical follow‐up, a therapist‐guided smartphone aftercare program did not improve outcomes beyond usual care. Beyond the specific outcome findings, the present trial provides important information for service development by suggesting that this type of low‐intensity digital aftercare intervention may not justify the additional time and resources required when no clear incremental benefit over usual care is observed. This is also relevant from a service perspective, because, despite digital delivery, the intervention still required individualized therapist guidance. Each feedback took approximately 25 min, representing a meaningful additional personnel investment. No formal health economic evaluation was conducted.

Future work should test blended models, longer support, and adaptive monitoring schedules, incorporate components targeting depressive symptoms, involve patients more closely in the design of aftercare interventions, and include male patients with AN in future trials.

## Author Contributions

**Sandra Schlegl:** conceptualization, methodology, investigation, funding acquisition, writing – original draft, project administration, supervision, data curation, validation. **Andreas Gerich:** writing – original draft, writing – review and editing, formal analysis, software, methodology, data curation, visualization, validation. **Yannik Terhorst:** writing – review and editing, formal analysis, software, methodology, data curation, validation. **Christina Fricke Neumayr:** conceptualization, writing – review and editing, investigation, methodology, validation. **Ulrich Voderholzer:** conceptualization, investigation, funding acquisition, writing – review and editing, supervision, resources, methodology, validation.

## Funding

This work is funded by a grant from the Swiss Anorexia Nervosa Foundation (Project #78‐17) to Sandra Schlegl and Ulrich Voderholzer. Funding sources did not have a role in the conceptualization, design, data collection, analysis, or interpretation of the data, writing the manuscript, or the decision to submit the paper for publication.

## Conflicts of Interest

The authors declare no conflicts of interest.

## Supporting information


**Table S1:** Post‐discharge healthcare utilization.
**Table S2:** Baseline characteristics by assessment completion status at 16 weeks.
**Table S3:** Baseline characteristics by assessment completion status at 6‐month follow‐up.
**Table S4:** Fixed effects estimates from linear mixed models for subscales of outcomes.
**Table S5:** Estimated marginal means and group comparisons across timepoints for subscales of outcomes.
**Table S6:** Within‐group changes from baseline to 16 weeks and 6‐month follow‐up.
**Table S7:** Fixed effects estimates from linear mixed models for sensitivity analysis for the Eating Disorder Examination Global Score.
**Table S8:** Estimated marginal means and group comparisons across timepoints for sensitivity analysis for the Eating Disorder Examination Global Score.

## Data Availability

The data that support the findings of this study are available from the corresponding author upon reasonable request.
