# Supplementary material for: Therapist‐Guided Smartphone‐Based Aftercare for Inpatients With Severe Anorexia Nervosa (SMART‐AN): A Randomized Clinical Trial
Source: Int J Eat Disord. 2026 Jun 23;59(10):2332–43. doi: 10.1002/eat.70142 (PMC13655992; doi:10.1002/eat.70142)
Supplement: Supplementary file 1 [file EAT-59-2332-s001.docx]

**Therapist-guided smartphone-based aftercare for inpatients**

**with severe anorexia nervosa (SMART-AN): A randomized clinical trial**

*Supplemental Materials*

Table S1: Post-discharge healthcare utilization

Table S2: Baseline characteristics by assessment completion status at 16 weeks

Table S3: Baseline characteristics by assessment completion status at 6-month follow-up

Table S4: Fixed effects estimates from linear mixed models for subscales of outcomes

Table S5: Estimated marginal means and group comparisons across timepoints for subscales of outcomes

Table S6: Within-group changes from baseline to 16 weeks and 6-month follow-up

Table S7: Fixed effects estimates from linear mixed models for sensitivity analysis for the Eating Disorder Examination Global Score

**Table S8: Estimated marginal means and group comparisons across timepoints for sensitivity analysis for the Eating Disorder Examination Global Score**

**Table S1: Post-discharge healthcare utilization**

|  | **Group** | **16 weeks**  IG (n = 59)  CG (n = 68)  Overall (N = 127) | **6-month FU**  IG (n = 55)  CG (n = 64)  Overall (N = 119) |
| --- | --- | --- | --- |
| **Outpatient psychotherapy**  *N (%)* | IG  CG | 53 (89.8)  64 (94.1) | 51 (92.7)  57 (86.4) |
|  | Overall | 117 (92.1) | 108 (89.3) |
| Number of treatment sessions since discharge  *M (SD)* | IG  CG | 16.53 (31.92)  12.53 (9.08) | 17.22 (10.57)  21.25 (24.70) |
|  | Overall | 14.34 (22.48) | 19.34 (19.38) |
| Time lag between discharge and the first outpatient treatment session after inpatient treatment in days  *M (SD)* | IG  CG | 2.23 (2.37)  2.84 (2.94) | --  -- |
|  | Overall | 2.57 (2.71) | -- |
| **Inpatient treatment**  *N (%)* | IG  CG | 5 (8.5)  1 (1.4) | 15 (27.3)  17 (25.8) |
|  | Overall | 6 (4.7) | 32 (26.4) |
| Number of treatments since discharge  *M (SD)* | IG  CG | 1.00 (0.00)  1.00 (n/a) | 1.78 (1.39)  1.29 (0.83) |
|  | Overall | 1.00 (0.00) | 1.48 (1.08) |
| **Day-care hospital**  *N* (%) | IG  CG | 1 (1.7)  0 (0.0) | 0 (0.0)  1 (1.4) |
|  | Overall | 1 (0.8) | 1 (0.7) |
| Number of treatments since discharge  *M (SD)* | IG  CG | 1.00 (n/a)  -- | --  1.00 (n/a) |
|  | Overall | 1.00 (n/a) | 1.00 (n/a) |
| **Psychiatrist**  *N (%)* | IG  CG | 29 (49.2)  21 (30.9) | 22 (40.0)  22 (33.3) |
|  | Overall | 50 (39.4) | 44 (36.4) |
| Number of appointments since discharge  *M (SD)* | IG  CG | 4.08 (5.25)  11.47 (7.61) | 5.77 (6.68)  15.57 (38.52) |
|  | Overall | 7.20 (7.28) | 10.56 (27.45) |
| **General practitioner**  *N (%)* | IG  CG | 36 (61.0)  32 (47.1) | 30 (54.6)  32 (48.5) |
|  | Overall | 68 (53.4) | 62 (51.2) |
| Number of appointments since discharge  *M (SD)* | IG  CG | 3.85 (3.96)  4.32 (4.76) | 5.57 (5.33)  4.52 (4.43) |
|  | Overall | 4.08 (4.33) | 5.03 (4.88) |
| **Nutrition counselling**  *N (%)* | IG  CG | 7 (11.9)  9 (13.2) | 4 (7.2)  7 (10.9) |
|  | Overall | 16 (12.6) | 11 (9.2) |
| Number of appointments since discharge  *M (SD)* | IG  CG | 3.29 (3.30)  2.88 (2.47) | 12.50 (9.61)  2.14 (1.57) |
|  | Overall | 3.07 (2.79) | 5.91 (7.52) |
| **Counseling center for eating disorders**  *N (%)* | IG  CG | 8 (13.6)  5 (7.4) | 6 (10.9)  5 (7.8) |
|  | Overall | 13 (10.2) | 11 (9.2) |
| Number of appointments since discharge  *M (SD)* | IG  CG | 2.57 (2.07)  2.25 (0.50) | 3.17 (1.17)  1.60 (0.89) |
|  | Overall | 2.09 (1.76) | 2.45 (1.29) |
| **General counseling center**  *N (%)* | IG  CG | 4 (6.8)  4 (5.9) | 1 (1.8)  2 (3.1) |
|  | Overall | 8 (6.3) | 3 (2.5) |
| Number of appointments since discharge  *M (SD)* | IG  CG | 1.75 (0.96)  2.33 (1.15) | 1.00 (n/a)  1.00 (0.00) |
|  | Overall | 2.00 (1.00) | 1.00 (0.00) |
| **Weight control**  *N* *(%)* | IG  CG | 34 (57.6)  38 (55.9) | 24 (43.6)  32 (48.5) |
|  | Overall | 72 (56.7) | 56 (46.3) |
| Number of appointments since discharge  *M (SD)* | IG  CG | 12.44 (8.60)  12.73 (8.00) | 8.63 (6.06)  14.13 (13.34) |
|  | Overall | 12.60 (8.23) | 11.68 (11.00) |
| **Therapeutic living community**  *N* *(%)* | IG  CG | 1 (1.7)  1 (1.5) | 2 (3.6)  0 (0.0) |
|  | Overall | 2 (1.6) | 2 (1.7) |
| **Self-help group**  *N (%)* | IG  CG | 0 (0.0)  2 (2.9) | 0 (0.0)  1 (1.6) |
|  | Overall | 2 (1.6) | 1 (0.8) |

*Note*: FU = Follow-Up, IG = Intervention Group, CG = Control Group, n/a = Not Applicable

**Table S2:** **Baseline characteristics by assessment completion status at 16 weeks**

| **Baseline variable** | **Completers**  **(n = 141)** | **Non-completers**  **(n = 45)** |
| --- | --- | --- |
| **Age** in years *M (SD),* *range* | 19.41 (6.44), 13-51 | 20.13 (6.84), 14-49 |
| **Adolescents** *n (%)* | 74 (52.5) | 21 (46.7) |
| **Weight-related characteristics** *M (SD)* |  |  |
| **BMI** in *kg/m^2^* (at admission) | 15.41 (1.62) | 15.24 (1.71) |
| **Percentile** (at admission) | 1.91 (4.92) | 1.41 (2.95) |
| **SDS** (at admission) | -2.98 (2.58) | -3.40 (1.60) |
| **BMI** in *kg/m^2^* (at randomization) | 18.96 (1.27) | 18.51 (1.39) |
| **Percentile** (at randomization) | 21.06 (15.08) | 16.58 (13.56) |
| **SDS** (at randomization) | -0.95 (0.64) | 1.21 (0.76) |
| **Minimal weight** in *kg* | 40.19 (5.80) | 38.79 (5.49) |
| **Minimal BMI** in *kg/m^2^* | 14.22 (2.05) | 14.24 (1.49) |
| **Maximal weight** in *kg* | 58.73 (8.35) | 60.03 (11.48) |
| **Maximal BMI** in *kg/m^2^* | 21.17 (4.79) | 21.50 (3.76) |
| **Weight suppression** *in kg* | 18.57 (6.94) | 20.24 (11.78) |
| **BMI severity (at admission)** *n (%)*  Mild  Moderate  Severe  Extreme | 23 (16.3)  29 (20.6)  34 (24.1)  55 (39.0) | 8 (17.8)  7 (15.6)  11 (24.4)  19 (42.2) |
| **BMI severity (at discharge)** *n (%)*  Mild  Moderate  Severe  Extreme | 133 (94.3)  5 (3.5)  3 (2.1)  0 (0.0) | 39 (86.7)  4 (8.9)  2 (4.4)  0 (0.0) |
| **Duration of ED** in years *M (SD)* | 3.49 (3.39) | 5.10 (6.37) |
| **Inpatient treatment duration** in days *M (SD)* | 125.51 (53.42) | 107.71 (44.42) |
| **Previous inpatient treatment** *M (SD)* | 1.24 (1.96) | 1.20 (1.36) |
| **Previous outpatient treatment** *M (SD)* | 1.24 (1.55) | 0.97 (0.72) |
| **Subtype of AN** *n (%)*  Restrictive type (F50.00)  Binge-eating/purging type (F50.01)  Atypical AN (F50.1) | 99 (70.7)  34 (24.3)  7 (5.0) | 29 (64.4)  14 (31.1)  2 (4.4) |
| **Any comorbid disorder** *n (%)* | 107 (75.9) | 32 (71.1) |
| **Comorbidity^†^** *n (%)*  Mild depressive episode (F32.0)  Moderate depressive episode (F32.1)  Severe depressive episode (F32.2)  Recurrent moderate depressive disorder (F33.1)  Recurrent severe depressive disorder (F33.2)  Social phobia (F40.1)  Posttraumatic stress disorder (F43.1) | 5 (3.5)  51 (36.2)  18 (12.8)  13 (9.2)  10 (7.1)  11 (7.8)  8 (5.7) | 3 (6.7)  15 (33.3)  4 (8.9)  6 (13.3)  2 (4.4)  2 (4.4)  1 (2.2) |
| **EDE** *M (SD)* |  |  |
| Global Score | 2.08 (1.16) | 1.80 (1.18) |
| Restraint | 1.29 (1.16) | 0.85 (0.83) |
| Eating Concern | 1.21 (1.04) | 0.99 (0.99) |
| Weight Concern | 2.12 (1.41) | 2.00 (1.50) |
| Shape Concern | 2.98 (1.53) | 2.75 (1.71) |
| **EDE-Q** *M (SD)* |  |  |
| Global Score | 2.20 (1.20) | 1.96 (1.33) |
| Restraint | 1.41 (1.20) | 1.01 (0.91) |
| Eating Concern | 1.42 (1.67) | 1.24 (1.12) |
| Weight Concern | 2.20 (1.53) | 2.03 (1.58) |
| Shape Concern | 3.16 (1.63) | 2.97 (1.92) |
| **BDI-II Global Score** *M (SD)* | 17.38 (12.94) | 17.57 (12.77) |
| **GSE Global Score** *M (SD)* | 25.29 (5.59) | 25.41 (6.31) |
| **SOCQ-ED Global Score** *M (SD)* | 62.11 (9.21) | 63.65 (8.91) |

*Note:* BMI = Body Mass Index, SDS = Standard Deviation Score, AN = Anorexia Nervosa, ED = Eating Disorder, EDE = Eating Disorder Examination, EDE-Q = Eating Disorder Examination-Questionnaire, BDI-II = Beck Depression Inventory-II, GSE = General Self-Efficacy Scale, SOCQ-ED = Stages of Change Questionnaire

† only diagnoses occurring in more than 5% of participants in either group are shown

**Table S3: Baseline characteristics by assessment completion status at 6-month follow-up**

| **Baseline variable** | **Completers**  **(n = 131)** | **Non-completers**  **(n = 55)** |
| --- | --- | --- |
| **Age** in years *M (SD),* *range* | 19.25 (6.37), 13-51 | 20.40 (6.87), 14-49 |
| **Adolescents** *n (%)* | 70 (53.4) | 25 (45.5) |
| **Weight-related characteristics** *M (SD)* |  |  |
| **BMI** in *kg/m^2^* (at admission) | 15.36 (1.62) | 15.41 (1.67) |
| **Percentile** (at admission) | 1.88 (5.02) | 1.58 (3.02) |
| **SDS** (at admission) | -2.99 (2.64) | -3.32 (1.61) |
| **BMI** in *kg/m^2^* (at randomization) | 18.94 (1.34) | 18.65 (1.25) |
| **Percentile** (at randomization) | 21.14 (15.20) | 17.19 (13.58) |
| **SDS** (at randomization) | -0.95 (0.65) | -1.15 (0.72) |
| **Minimal weight** in *kg* | 40.16 (5.87) | 39.95 (5.36) |
| **Minimal BMI** in *kg/m^2^* | 14.14 (2.09) | 14.44 (1.46) |
| **Maximal weight** in *kg* | 58.58 (7.75) | 60.17 (12.00) |
| **Maximal BMI** in *kg/m^2^* | 20.74 (2.45) | 22.58 (7.68) |
| **Weight suppression** *in kg* | 18.44 (7.05) | 20.48 (10.52) |
| **BMI severity (at admission)** *n (%)*  Mild  Moderate  Severe  Extreme | 22 (16.8)  23 (17.6)  32 (24.4)  54 (41.2) | 9 (16.4)  13 (23.6)  13 (23.6)  20 (36.4) |
| **BMI severity (at discharge)** *n (%)*  Mild  Moderate  Severe  Extreme | 122 (93.1)  6 (4.6)  3 (2.3)  0 (0.0) | 50 (90.9)  3 (5.4)  2 (3.6)  0 (0.0) |
| **Duration of ED** in years *M (SD)* | 3.49 (3.47) | 4.83 (5.88) |
| **Inpatient treatment duration** in days *M (SD)* | 125.57 (53.25) | 110.82 (47.14) |
| **Previous inpatient treatment** *M (SD)* | 1.25 (1.61) | 1.00 (0.66) |
| **Previous outpatient treatment** *M (SD)* | 1.27 (1.98) | 1.15 (1.41) |
| **Subtype of AN** *n (%)*  Restrictive type (F50.00)  Binge-eating/purging type (F50.01)  Atypical AN (F50.1) | 97 (74.6)  26 (19.8)  7 (5.3) | 31 (56.4)  22 (40.0)  2 (3.6) |
| **Any comorbid disorder** *n (%)* | 97 (74.0) | 42 (76.4) |
| **Comorbidity^†^** *n (%)*  Mild depressive episode (F32.0)  Moderate depressive episode (F32.1)  Severe depressive episode (F32.2)  Recurrent moderate depressive disorder (F33.1)  Recurrent severe depressive disorder (F33.2)  Social phobia (F40.1)  Mixed obsessional thoughts and acts (F42.2)  Posttraumatic stress disorder (F43.1) | 5 (3.8)  49 (37.4)  16 (12.2)  9 (6.9)  9 (6.9)  9 (6.9)  5 (3.8)  8 (6.1) | 3 (5.5)  17 (30.9)  6 (10.9)  10 (18.2)  3 (5.5)  4 (7.3)  3 (5.5)  1 (1.8) |
| **EDE** *M (SD)* |  |  |
| Global Score | 2.03 (1.18) | 1.97 (1.17) |
| Restraint | 1.27 (1.15) | 0.97 (0.96) |
| Eating Concern | 1.19 (1.05) | 1.07 (0.98) |
| Weight Concern | 2.05 (1.40) | 2.19 (1.49) |
| Shape Concern | 2.91 (1.52) | 2.97 (1.69) |
| **EDE-Q** *M (SD)* |  |  |
| Global Score  Restraint | 2.13 (1.28)  1.37 (1.18) | 2.91 (1.30)  1.23 (1.10) |
| Eating Concern | 1.39 (1.16) | 1.37 (1.17) |
| Weight Concern | 2.11 (1.56) | 2.30 (1.50) |
| Shape Concern | 3.09 (1.64) | 3.22 (1.83) |
| **BDI-II Global Score** *M (SD)* | 16.92 (12.65) | 18.79 (13.47) |
| **GSE Global Score** *M (SD)* | 25.23 (5.77) | 25.53 (5.68) |
| **SOCQ-ED Global Score** *M (SD)* | 62.70 (9.11) | 61.74 (9.29) |

*Note:* BMI = Body Mass Index, SDS = Standard Deviation Score, AN = Anorexia Nervosa, ED = Eating Disorder, EDE = Eating Disorder Examination, EDE-Q = Eating Disorder Examination-Questionnaire, BDI-II = Beck Depression Inventory-II, GSE = General Self-Efficacy Scale, SOCQ-ED = Stages of Change Questionnaire for Eating Disorders

† only diagnoses occurring in more than 5% of participants in either group are shown

**Table S4: Fixed effects estimates from linear mixed models for subscales of outcomes**

| **Outcome** | **Fixed effects** | **Estimate** | **SE** | **df** | **t value** | ***p* value** |
| --- | --- | --- | --- | --- | --- | --- |
| EDE Restraint | Intercept | 1.25 | 0.13 | 297.83 | 9.31 | <.001*** |
|  | Timepoint | 0.04 | 0.02 | 308.16 | 2.24 | .026* |
|  | Group | 0.05 | 0.19 | 298.78 | 0.28 | .780 |
|  | Timepoint:Group | 0.02 | 0.02 | 310.46 | 0.72 | .470 |
| EDE Eating Concern | Intercept | 1.28 | 0.11 | 276.01 | 11.04 | <.001*** |
|  | Timepoint | 0.01 | 0.01 | 299.90 | 1.04 | .297 |
|  | Group | -0.05 | 0.16 | 275.97 | -0.28 | .783 |
|  | Timepoint:Group | 0.04 | 0.02 | 301.44 | 1.94 | .054 |
| EDE Weight Concern | Intercept | 2.09 | 0.15 | 252.30 | 13.83 | <.001*** |
|  | Timepoint | -0.03 | 0.02 | 290.02 | -1.73 | .085 |
|  | Group | 0.01 | 0.21 | 253.02 | 0.03 | .973 |
|  | Timepoint:Group | 0.04 | 0.02 | 291.53 | 1.58 | .114 |
| EDE Shape Concern | Intercept | 2.84 | 0.16 | 235.66 | 17.74 | <.001*** |
|  | Timepoint | -0.05 | 0.02 | 283.46 | -3.50 | <.001*** |
|  | Group | 0.09 | 0.23 | 234.93 | 0.38 | .707 |
|  | Timepoint:Group | 0.02 | 0.02 | 283.96 | 1.14 | .253 |
| EDE-Q Restraint | Intercept | 1.41 | 0.14 | 264.99 | 9.90 | <.001*** |
|  | Timepoint | 0.06 | 0.02 | 276.00 | 3.62 | <.001*** |
|  | Group | 0.04 | 0.20 | 267.17 | 0.22 | .829 |
|  | Timepoint:Group | 0.03 | 0.02 | 279.83 | 1.25 | .211 |
| EDE-Q Eating Concern | Intercept | 1.41 | 0.13 | 227.30 | 10.82 | <.001*** |
|  | Timepoint | 0.03 | 0.01 | 262.73 | 2.08 | .038 |
|  | Group | 0.06 | 0.19 | 228.88 | 0.32 | .748 |
|  | Timepoint:Group | 0.03 | 0.02 | 265.26 | 1.86 | .065 |
| EDE-Q Weight Concern | Intercept | 2.10 | 0.17 | 239.51 | 12.38 | <.001*** |
|  | Timepoint | 0.00 | 0.02 | 266.54 | 0.10 | .916 |
|  | Group | 0.21 | 0.24 | 241.35 | 0.89 | .375 |
|  | Timepoint:Group | 0.03 | 0.03 | 269.56 | 1.22 | .224 |
| EDE-Q Shape Concern | Intercept | 2.98 | 0.18 | 212.81 | 16.86 | <.001*** |
|  | Timepoint | -0.02 | 0.02 | 255.70 | -1.56 | .121 |
|  | Group | 0.24 | 0.25 | 214.13 | 0.96 | .336 |
|  | Timepoint:Group | 0.02 | 0.02 | 257.79 | 0.99 | .325 |

*Note*: SE = Standard Error, df = Degrees of Freedom, EDE = Eating Disorder Examination, EDE-Q = Eating Disorder Examination-Questionnaire, BMI = Body Mass Index, BDI-II = Beck Depression Inventory-II, GSE = General Self-Efficacy Scale, SOCQ-ED = Stages of Change Questionnaire for Eating Disorders, ** p* <.05, ** *p* <.01, *** *p* <.001

**Table S5: Estimated marginal means and group comparisons across timepoints for subscales of outcomes**

| **Outcome** | **Timepoint** | **Intervention Group**  **EMM (95% CI) [n]** | **Control Group**  **EMM (95% CI) [n]** | **Difference in LS means (95% CI)** | ***p* value** | **Effect size**  **(95% CI)** |
| --- | --- | --- | --- | --- | --- | --- |
| EDE Restraint | Baseline | 1.30 (1.04-1.57) [93] | 1.25 (0.99-1.52) [93] | 0.05 (-0.32-0.43) | .780 | 0.05 (-0.32-0.43) |
|  | 16 weeks | 1.52 (1.28-1.76) [69] | 1.40 (1.16-1.63) [72] | 0.12 (-0.21-0.45) | .471 | 0.12 (-0.21-0.45) |
|  | 6-month FU | 1.84 (1.53-2.16) [63] | 1.62 (1.31-1.93) [68] | 0.22 (-0.22-0.66) | .317 | 0.22 (-0.22-0.66) |
| EDE Eating Concern | Baseline | 1.23 (1.00-1.46) [93] | 1.28 (1.05-1.50) [92] | -0.05 (-0.37-0.28) | .783 | -0.06 (-0.46-0.35) |
|  | 16 weeks | 1.43 (1.23-1.64) [69] | 1.33 (1.13-1.54) [72] | 0.10 (-0.19-0.39) | .494 | 0.13 (-0.24-0.49) |
|  | 6-month FU | 1.73 (1.47-2.00) [63] | 1.41 (1.15-1.67) [68] | 0.32 (-0.05-0.69) | .091 | 0.40 (-0.07-0.87) |
| EDE Weight Concern | Baseline | 2.09 (1.80-2.39) [93] | 2.09 (1.79-2.38) [93] | 0.01 (-0.41-0.43) | .973 | 0.01 (-0.43-0.45) |
|  | 16 weeks | 2.13 (1.85-2.40) [69] | 1.98 (1.70-2.25) [72] | 0.15 (-0.23-0.54) | .438 | 0.16 (-0.24-0.56) |
|  | 6-month FU | 2.18 (1.84-2.52) [63] | 1.81 (1.47-2.15) [67] | 0.37 (-0.11-0.85) | .130 | 0.39 (-0.11-0.89) |
| EDE Shape Concern | Baseline | 2.93 (2.61-3.24) [93] | 2.84 (2.53-3.16) [91] | 0.09 (-0.36-0.53) | .707 | 0.09 (-0.40-0.59) |
|  | 16 weeks | 2.81 (2.52-3.11) [69] | 2.63 (2.33-2.93) [72] | 0.18 (-0.23-0.60) | .385 | 0.20 (-0.26-0.67) |
|  | 6-month FU | 2.64 (2.29-3.00) [63] | 2.31 (1.96-2.66) [67] | 0.33 (-0.16-0.83) | .187 | 0.37 (-0.18-0.92) |
| EDE-Q Restraint | Baseline | 1.45 (1.16-1.74) [86] | 1.41 (1.13-1.69) [89] | 0.04 (-0.36-0.44) | .829 | 0.04 (-0.37-0.46) |
|  | 16 weeks | 1.81 (1.55-2.07) [59] | 1.64 (1.39-1.89) [68] | 0.16 (-0.20-0.53) | .370 | 0.17 (-0.20-0.54) |
|  | 6-month FU | 2.34 (2.00-2.68) [55] | 2.00 (1.68-2.32) [66] | 0.35 (-0.12-0.81) | .147 | 0.35 (-0.13-0.83) |
| EDE-Q Eating Concern | Baseline | 1.47 (1.21-1.74) [86] | 1.41 (1.16-1.67) [89] | 0.06 (-0.31-0.43) | .748 | 0.08 (-0.42-0.58) |
|  | 16 weeks | 1.72 (1.47-1.96) [59] | 1.52 (1.28-1.76) [68] | 0.20 (-0.15-0.54) | .262 | 0.27 (-0.20-0.73) |
|  | 6-month FU | 2.08 (1.78-2.38) [55] | 1.67 (1.39-1.96) [66] | 0.40 (-0.01-0.82) | .058 | 0.54 (-0.02-1.11) |
| EDE-Q Weight Concern | Baseline | 2.29 (1.95-2.62) [86] | 2.07 (1.74-2.40) [89] | 0.21 (-0.26-0.68) | .375 | 0.21 (-0.25-0.67) |
|  | 16 weeks | 2.42 (2.10-2.73) [59] | 2.08 (1.78-2.38) [68] | 0.34 (-0.10-0.77) | .129 | 0.33 (-0.10-0.75) |
|  | 6-month FU | 2.61 (2.22-3.01) [55] | 2.09 (1.72-2.46) [66] | 0.52 (-0.02-1.06) | .057 | 0.51 (-0.02-1.04) |
| EDE-Q Shape Concern | Baseline | 3.22 (2.87-3.58) [86] | 2.98 (2.63-3.33) [89] | 0.24 (-0.25-0.74) | .336 | 0.27 (-0.28-0.82) |
|  | 16 weeks | 3.22 (2.88-3.56) [59] | 2.88 (2.55-3.21) [68] | 0.33 (-0.14-0.81) | .165 | 0.37 (-0.15-0.89) |
|  | 6-month FU | 3.21 (2.81-3.61) [55] | 2.74 (2.36-3.12) [66] | 0.47 (-0.08-1.02) | .096 | 0.51 (-0.09-1.12) |

*Note*: EMM = Estimated Marginal Means, CI = Confidence Interval, LS = Least Squares, EDE = Eating Disorder Examination, EDE-Q = Eating Disorder Examination-Questionnaire, FU = Follow-Up

**Table S6: Within-group changes from baseline to 16 weeks and 6-month follow-up**

|  | |  | | **Within group mean change**  **M (SD)** | | | | **Effect size**  **(95% CI)** | | | |
| --- | --- | --- | --- | --- | --- | --- | --- | --- | --- | --- | --- |
|  | |  | | **Intervention Group** | | **Control Group** | | **Intervention Group** | | **Control Group** | |
| **Primary Outcome** | | | |  | |  | |  | |  | |
| EDE Global Score | | 16 weeks | | 0.03 (0.93) | | 0.08 (0.93) | | 0.03 (-0.20-0.27) | | 0.08 (-0.15-0.32) | |
|  | | 6-month FU | | 0.08 (1.21) | | -0.09 (1.12) | | 0.06 (-0.19-0.31) | | -0.08 (-0.32-0.17) | |
| **Secondary Outcomes** | | | |  | |  | |  | |  | |
| EDE Restraint | | 16 weeks | | 0.40 (1.35) | | 0.47 (1.18) | | 0.30 (0.06-0.54)* | | 0.40 (0.16-0.64)** | |
|  | | 6-month FU | | 0.44 (1.65) | | 0.38 (1.45) | | 0.26 (0.01-0.51)* | | 0.26 (0.02-0.50)* | |
| EDE Eating Concern | | 16 weeks | | 0.50 (0.93) | | 0.40 (1.06) | | 0.53 (0.28-0.79)*** | | 0.38 (0.14-0.62)** | |
|  | | 6-month FU | | 0.50 (1.20) | | 0.15 (1.16) | | 0.41 (0.16-0.67)** | | 0.13 (-0.11-0.37) | |
| EDE Weight Concern | | 16 weeks | | -0.11 (1.25) | | -0.02 (1.35) | | -0.09 (-0.32-0.15) | | -0.02 (-0.25-0.22) | |
|  | | 6-month FU | | 0.09 (1.51) | | -0.22 (1.39) | | 0.06 (-0.19-0.31) | | -0.16 (-0.40-0.08) | |
| EDE Shape Concern | | 16 weeks | | -0.36 (1.18) | | -0.23 (1.21) | | -0.31 (-0.55--0.06)* | | -0.19 (-0.43-0.04) | |
|  | | 6-month FU | | -0.30 (1.39) | | -0.47 (1.35) | | -0.21 (-0.46-0.04) | | -0.35 (-0.60--0.10)** | |
| EDE-Q Global Score | | 16 weeks | | 0.18 (1.02) | | 0.28 (1.07) | | 0.18 (-0.08-0.44) | | 0.26 (0.02-0.51)* | |
|  | | 6-month FU | | 0.37 (1.33) | | 0.12 (1.11) | | 0.28 (0.01-0.55)* | | 0.11 (-0.13-0.35) | |
| EDE-Q Restraint | | 16 weeks | | 0.56 (1.23) | | 0.69 (1.20) | | 0.45 (0.18-0.72)** | | 0.57 (0.32-0.83)*** | |
|  | | 6-month FU | | 0.84 (1.67) | | 0.61 (1.46) | | 0.50 (0.22-0.78)** | | 0.41 (0.16-0.67)** | |
| EDE-Q Eating Concern | | 16 weeks | | 0.27 (1.01) | | 0.50 (0.90) | | 0.27 (0.01-0.53)* | | 0.55 (0.29-0.81)*** | |
|  | | 6-month FU | | 0.57 (1.12) | | 0.28 (0.99) | | 0.51 (0.22-0.79)** | | 0.28 (0.03-0.53)* | |
| EDE-Q Weight Concern | | 16 weeks | | 0.08 (1.31) | | 0.23 (1.52) | | 0.06 (-0.19-0.32) | | 0.15 (-0.09-0.39) | |
|  | | 6-month FU | | 0.34 (1.68) | | 0.05 (1.42) | | 0.20 (-0.07-0.47) | | 0.04 (-0.20-0.28) | |
| EDE-Q Shape Concern | | 16 weeks | | -0.04 (1.26) | | -0.08 (1.28) | | -0.03 (-0.29-0.23) | | -0.06 (-0.30-0.18) | |
|  | | 6-month FU | | -0.20 (1.54) | | -0.23 (1.24) | | -0.01 (-0.28-0.25) | | -0.18 (-0.43-0.06) | |
| BMI | | 16 weeks | | -0.51 (1.86) | | -0.51 (1.50) | | -0.28 (-0.55-0.00)* | | -0.34 (-0.61--0.07)* | |
|  | | 6-month FU | | -0.63 (1.90) | | -0.59 (1.66) | | -0.33 (-0.61--0.06)* | | -0.36 (-0.63--0.08)* | |
| BDI-II Global Score | | 16 weeks | | 4.50 (9.60) | | 5.12 (8.12) | | 0.47 (0.20-0.74)** | | 0.63 (0.36-0.89)*** | |
|  | | 6-month FU | | 6.98 (11.71) | | 5.20 (9.60) | | 0.60 (0.31-0.89)*** | | 0.54 (0.28-0.80)*** | |
| GSE Global Score | | 16 weeks | | -0.72 (4.51) | | -1.64 (4.09) | | -0.16 (-0.42-0.10) | | -0.40 (-0.65--0.15)** | |
|  | | 6-month FU | | -1.24 (6.24) | | -1.21 (4.90) | | -0.20 (-0.47-0.07) | | -0.25 (-0.49-0.00)* | |
| SOCQ-ED Global Score | | 16 weeks | | -3.07 (7.64) | | -3.96 (9.00) | | -0.40 (-0.67--0.13)** | | -0.44 (-0.69--0.19)** | |
|  | 6-month FU | | -3.89 (11.71) | | -2.55 (10.22) | | -0.33 (-0.61--0.06)* | | -0.25 (-0.49-0.00)* | |  |

*Note:* CI = Confidence Interval, EDE = Eating Disorder Examination, FU = Follow-Up, EDE-Q = Eating Disorder Examination-Questionnaire, BMI = Body Mass Index, BDI-II = Beck Depression Inventory-II, GSE = General Self-Efficacy Scale, SOCQ-ED = Stages of Change Questionnaire for Eating Disorders, ** p* < .05, ** *p* < .01, *** *p* < .001

**Table S7: Fixed effects estimates from linear mixed models for sensitivity analysis for the Eating Disorder Examination Global Score**

| **Outcome** | **Fixed effects** | **Estimate** | **SE** | **df** | **t value** | ***p* value** | **RIV** | **FMI** |
| --- | --- | --- | --- | --- | --- | --- | --- | --- |
| **Multiple Imputation** |  |  |  |  |  |  |  |  |
|  | Intercept | 2.00 | 0.13 | 12489.40 | 15.53 | <.001 | 0.06 | 0.06 |
|  | Timepoint | -0.01 | 0.01 | 84.99 | -0.86 | .392 | 0.48 | 0.34 |
|  | Group | 0.05 | 0.18 | 4581.41 | 0.26 | .798 | 0.05 | 0.05 |
|  | Timepoint:Group | -0.02 | 0.02 | 259.76 | 1.00 | .318 | 0.23 | 0.19 |
| **Per-protocol** |  |  |  |  |  |  |  |  |
|  | Intercept | 2.02 | 0.12 | 216.79 | 16.16 | <.001 |  |  |
|  | Timepoint | -0.01 | 0.01 | 266.47 | -1.13 | .258 |  |  |
|  | Group | 0.03 | 0.19 | 212.30 | 0.14 | .889 |  |  |
|  | Timepoint:Group | 0.03 | 0.02 | 261.05 | 1.43 | .152 |  |  |
| **≥12-week adherence sample** |  |  |  |  |  |  |  |  |
|  | Intercept | 2.02 | 0.12 | 224.56 | 16.24 | <.001 |  |  |
|  | Timepoint | -0.01 | 0.01 | 272.98 | -1.13 | .258 |  |  |
|  | Group | 0.01 | 0.19 | 220.63 | 0.06 | .955 |  |  |
|  | Timepoint:Group | 0.03 | 0.02 | 268.42 | 1.40 | .163 |  |  |

*Note*: SE = Standard Error, df = Degrees of Freedom, RIV = Relative Increase in Variance, FMI = Fraction of Missing Information

**Table S8: Estimated marginal means and group comparisons across timepoints for sensitivity analysis for the Eating Disorder Examination Global Score**

|  | **Timepoint** | **Intervention**  **Group**  **EMM**  **(95% CI)** | **Control**  **Group**  **EMM**  **(95% CI)** | **Difference in LS means**  **(95% CI)** | ***p* value** | **Effect size**  **(95% CI)** |
| --- | --- | --- | --- | --- | --- | --- |
| **Multiple Imputation** |  |  |  |  |  |  |
|  | Baseline | 2.05  (1.80-2.29) | 2.00  (1.75-2.25) | -0.05  (-0.40-0.31) | .798 | -0.06  (-0.53-0.40) |
|  | 16 weeks | 2.07  (1.84-2.29) | 1.95  (1.73-2.18) | 0.12  (-0.44-0.21) | .488 | -0.15  (-0.58-0.28) |
|  | 6-month FU | 2.10  (1.85-2.36) | 1.88  (1.63-2.14) | -0.22  (-0.61-0.17) | .266 | -0.29  (-0.80-0.22) |
| **Per-protocol** |  |  |  |  |  |  |
|  | Baseline | 2.05  (1.76-2.34) | 2.02  (1.78-2.27) | 0.03  (-0.36-0.41) | .889 | 0.04  (-0.48-0.56) |
|  | 16 weeks | 2.10  (1.83-2.37) | 1.97  (1.74-2.20) | 0.13  (-0.22-0.49) | .456 | 0.18  (-0.30-0.67) |
|  | 6-month FU | 2.18  (1.87-2.49) | 1.88  (1.61-2.16) | 0.29  (-0.12-0.71) | .165 | 0.40  (-0.17-0.97) |
| **≥12-week adherence sample** |  |  |  |  |  |  |
|  | Baseline | 2.04  (1.76-2.31) | 2.02  (1.78-2.27) | 0.01  (-0.36-0.38) | .955 | 0.01  (-0.49-0.52) |
|  | 16 weeks | 2.08  (1.82-2.34) | 1.97  (1.74-2.20) | 0.11  (-0.23-0.46) | .516 | 0.15  (-0.31-0.62) |
|  | 6-month FU | 2.15  (1.85-2.45) | 1.88  (1.61-2.16) | 0.27  (-0.14-0.67) | .197 | 0.36  (-0.19-0.92) |

*Note*: EMM = Estimated Marginal Means, LS = Least Squares, CI = Confidence Interval, FU = Follow-Up
